# Exploring the ‘train the trainer’ model for delivering Making Every Contact Count (MECC) training at scale: A qualitative study

**DOI:** 10.1111/bjhp.70076

**Published:** 2026-04-26

**Authors:** Beth Nichol, Angela M. Rodrigues, Sarah Audsley, Anna Haste, Mei Yee Tang, Craig Robson, Jill Harland, Catherine Haighton

**Affiliations:** ^1^ NIHR Policy Research Unit in Behavioural and Social Sciences, Newcastle University, UK; Population Health Sciences Institute, Faculty of Medical Sciences, Newcastle University Newcastle upon Tyne UK; ^2^ School of Communities and Education, Faculty of Health and Wellbeing, Northumbria University Newcastle upon Tyne UK; ^3^ Department of Psychology, Faculty of Health and Wellbeing Northumbria University Newcastle upon Tyne UK; ^4^ Fuse, Centre for Translational Research in Public Health Newcastle University Newcastle upon Tyne UK; ^5^ Department of Sport, Exercise and Rehabilitation, Faculty of Health and Wellbeing Northumbria University Newcastle upon Tyne UK; ^6^ School of Social Sciences, Humanities & Law, Teesside University Middlesbrough UK; ^7^ School of Psychology, University of Leeds Leeds UK; ^8^ Northumbria Healthcare NHS Foundation Trust Newcastle upon Tyne UK

**Keywords:** cascade, education, Making Every Contact Coun, opportunistic health interventions, qualitative, Theoretical Domains Framework, train the trainer

## Abstract

**Objectives:**

To explore the delivery of the train the trainer (TtT) model for Making Every Contact Count (MECC) training in the North East and North Cumbria (NENC) region of England.

**Design:**

A qualitative study, utilizing semi‐structured interviews.

**Methods:**

Interviews were conducted with 21 participants, including MECC TtT trainees (*n* = 13), eligible non‐trainees (*n* = 6) and principal trainers (*n* = 2). Data analysis utilized the Framework Method guided by the Theoretical Domains Framework (TDF), and meta‐themes were generated that transcended individual TDF domains.

**Results:**

Four meta‐themes were identified: the need for psychological preparedness to deliver MECC training, successful cascade is influenced by the MECC training content, limited accessibility of the pedagogical approach to both MECC TtT and MECC training, and a need to shift the focus from quantity to quality of MECC training cascade.

**Conclusions:**

The TtT model potentially provides unique value for delivering high‐quality MECC training at scale, providing trainers feel ownership over and able to deliver MECC training. A skills‐based approach to MECC training and an experiential learning approach to MECC TtT training are recommended. The MECC TtT programme should provide clear expectations of cascade at sign up stage, allow trainers to adapt content and evaluate success based on quality.

**Statement of Contribution**

**What is already known on this subject?**

- There is a high demand for models that can deliver training in Making Every Contact Count (MECC) at scale.
- The train the trainer model offers unique benefits including efficient dissemination of training.

**What does this study add?**

- Trainers need to feel a sense of ownership over MECC training to cascade it.
- Trainers need to feel confident and competent in delivering the training content to cascade it.
- ‘Success’ of the model should be judged through the quality of MECC training, not quantity.

## INTRODUCTION

Making Every Contact Count is a person‐centred approach to behaviour change conversations to promote health and wellbeing (Nichol et al., 2024). More evidence is needed to demonstrate a clear effectiveness of MECC on improvements in health and wellbeing of service users (Adam et al., 2020; Baird et al., 2014; Parchment et al., 2021), although evidence is promising to suggest that MECC is acceptable amongst service providers (Lawrence et al., 2016; Parchment et al., 2022; Watson et al., 2020) and users (Lawrence, Vogel, et al., 2020; Parchment et al., 2023). MECC also compliments the aims of key policy documents in the UK (Department of Health SS, Safety P, 2011; National Health Service N, 2014; Scotland N, 2013; Wales N, 2011) and internationally (WHO, 2015) by contributing to a wider culture change towards health promotion and disease prevention (Harrison et al., 2022; Mills, 2019; Parchment et al., 2021; Rodrigues et al., 2024). For example, as part of a holistic and person‐centred approach to population health as endorsed by the World Health Organization (WHO) (WHO, 2015), individuals are recognized as active agents with the ability to make choices relating to their own health, including engaging in positive choices described as self‐care (World Health Organization W, n.d.). Subsequently, the WHO recognizes the importance of self‐care in health promotion and disease prevention on a global scale (World Health Organization W, n.d.). A consistent message of health promotion through empowerment to engage in self‐care behaviours across sectors, for example by applying MECC, may offer the highest probability of health behaviour change and ultimately a reduction in non‐communicable diseases on a population level.

MECC was initially proposed by NHS England (Public Health England P, 2016) within a consensus statement as an approach to mitigate the rise in non‐communicable diseases including cardiovascular disease and cancers through discussing the drivers of smoking, alcohol consumption, lack of physical activity and poor diet (Organization WH, 2021). More recently, MECC has been applied to behaviour change more holistically to include a wide range of behaviours relevant to the individual (Chisholm et al., 2020; Parchment et al., 2022), in recognition of the wide scope of determinants of health and wellbeing (Dahlgren & Whitehead, 1991). Indeed, a recent consensus study of 40 experts in MECC confirmed that MECC is not defined by the target behaviour, topic of conversation or even who it is delivered by and where, and instead is defined by its person‐centred and motivational approach (Nichol et al., 2024). Therefore, it is conceivable that the potential scope and relevance of MECC is far‐reaching, beyond health care where it was initially implemented. However, disseminating the relevant skills and knowledge required to deliver MECC across multiple sectors presents a challenge due to limited resources (Rodrigues et al., 2024).

The need for a culture change towards prevention and self‐care combined with the wide potential scope of MECC has created a demand for the delivery of MECC training at scale. Subsequently, although regional approaches to the delivery of MECC training vary (Rodrigues et al., 2024; Tinati et al., 2012; Turner et al., 2023), many adopt a ‘Train the Trainer’ (TtT) model to deliver their MECC training (Hollis et al., 2022; Rodrigues et al., 2024; Turner et al., 2023). The TtT model involves using a principal trainer to provide training to individuals so that they become MECC trainers, cascading MECC training to those they go on to deliver training to.

The TtT model is an established strategic approach within the wider education literature including health education, given that it is proposed as cost‐effective (El‐Hamamsy et al., 2024; Harper et al., 2024), can deliver training at scale (El‐Hamamsy et al., 2024; Tobias et al., 2012; Yarber et al., 2015) and facilitates delivery of training that is sensitive to the context it is delivered in through opportunities for tailoring (El‐Hamamsy et al., 2024; Harper et al., 2024; Hayes, 2000; Yarber et al., 2015). Whilst there are well‐documented concerns within the wider education literature that a TtT model leads to dilution of key messages (El‐Hamamsy et al., 2024), empirical findings from health education literature indicate that the quality of the training that is cascaded is at least equal to other models (Lang et al., 2017; Lloyd et al., 2009; Martino et al., 2011; Nexø et al., 2024; Poitras et al., 2021), in terms of its ability to increase knowledge, motivation, intentions and delivery of the target behaviour (Nexø et al., 2024; Pearce, Mann, et al., 2012; Trabeau et al., 2008). Furthermore, one study found that the TtT model led to comparatively greater outcomes related to adherence and competence of the target behaviour compared with training delivered by an expert (Fitzsimmons‐Craft et al., 2021). When successful, the TtT model thus offers a wider potential reach of health training, of the same quality, with the same amount of resources (Tobias et al., 2012; Yarber et al., 2015). Within the context of MECC, given that the wide scope of MECC means that individuals from any role or setting could receive MECC training, the TtT model means that MECC trainers are more likely to possess specific knowledge of their audience, increasing its perceived relevance (Tobias et al., 2012; Yarber et al., 2015). However, TtT models often report a low cascade rate of training (Harper et al., 2024) (with as few as 20% of trainers cascading training (Orfaly et al., 2005)), upon which the success of the TtT model depends. Barriers within the wider education literature include a lack of confidence of trainers (El‐Hamamsy et al., 2024; Lloyd et al., 2009; Orfaly et al., 2005) and the time and planning required to organize and conduct training (El‐Hamamsy et al., 2024; Orfaly et al., 2005). Despite the increasing utilization of the TtT model to deliver MECC training (Hollis et al., 2022; Rodrigues et al., 2024), there lacks a qualitative evaluation to better understand the barriers and facilitators to implementing the TtT model. Given the early stage of research, qualitative analysis is appropriate to explore the experiences of individuals who have received MECC TtT training, the challenges they faced in cascading training and their thoughts on the suitability of the TtT model for the delivery of MECC training. Thus, this study reports the in‐depth qualitative analysis of the second stage of a mixed methods sequential study that evaluated the TtT model of MECC training across the North East and North Cumbria (NENC) region of England, reported elsewhere (Nichol et al., 2026). The aim of this stage was to explore the in‐depth explanations for the low rate of cascade of MECC training identified in stage one.

## METHODS

### Study design and setting

The current study describes the themes generated from a qualitative analysis of interview and survey data collected as part of the wider study (Nichol et al., 2026). The ontology for this study was critical realist, accepting an objective reality that is only subjectively perceived by individuals (Bhaskar, 2010), although a pragmatist epistemological position was adopted which selected the most appropriate method to address the behavioural problem (Mounce, 2002). Namely, the study applied behavioural science to provide a framework for barriers and enablers to training cascade. Namely, the Theoretical Domains Framework (TDF) was applied which divides behavioural determinants into 14 domains corresponding to capability, opportunity, or motivation to perform a specified behaviour (Cane et al., 2012). Reporting of the following methods adhered to the Consolidated criteria for reporting qualitative research (COREQ, see Data S1) (Tong et al., 2007), with the caveat that information power was selected as a more appropriate and valid sample size justification than data saturation (see further explanation below) (Malterud et al., 2016). As part of the wider mixed methods study, a professional contributor panel of four contributors was formed to advise on the study (more details are provided in the mixed methods paper: (Nichol et al., 2026)). Prior to data collection, the study protocol for the full mixed methods study was preregistered via Open Science Framework (https://osf.io/xz8au).

This study was approved by the Ethics department at Northumbria University (Project ID 5033). This study also received R&D approval from the North East and North Cumbria Integrated Care System (ICS) as a service evaluation, as determined by the Health Research Authority.

### Participants and recruitment

To explore potential barriers to accessing MECC TtT training, interview participants (*n* = 21) were eligible for MECC TtT training but may not have received it. Participants were adults (≥ 18) residing in the UK. Sampling was purposive to ensure that the sample was representative of current implementation of the TtT programme in terms of sectors (e.g., health care, local authority, third sector), level of training delivery or uptake (including those who had attended and cascaded training and those who had not) and type of participation in training (self‐referred or delegated to attend). The sample was organized into three distinct groups: principal trainers (*n* = 2), those who completed the MECC TtT programme (*n* = 13) and those who were eligible but had not completed the TtT programme (*n* = 6). Of those who had completed the programme, two participants were also involved in the implementation of MECC TtT and thus were also able to provide insights on the success of others in becoming trainers.

The Regional Delivering MECC at Scale Coordinator for the NENC and the MECC at Scale TtT course trainer (principal trainer) were asked to provide eligible participants with the study information sheet and expression of interest form which potential participants could complete via a Qualtrics (Qualtrics, 2013) link (Qualtrics, 2013). The Regional Delivering MECC at Scale Coordinator utilized existing networks and contacted potential participants via email, within online Teams meetings (e.g., MECC strategy group), and in‐person (e.g., at TtT sessions). When recruitment occurred in‐person, expression of interest forms were available through paper copies. Recruitment also took place via alternative routes such as third sector networks and existing individual contacts using the same approach. To facilitate purposive sampling, expression of interest forms collected optional basic demographic data (occupation, setting and receipt of MECC training). In addition, the principal trainer participated in an interview. Thus, recruitment occurred on an opt‐in basis.

Sample size for qualitative interviews was defined apriori using the model of information power by Malterud, Siersma and Guassora (Malterud et al., 2016). Target sample size was estimated from the aim, specificity of sample, use of theory, interviews and analysis strategy. As the aim was relatively specific, sampling was purposive, analysis used an established theoretical framework and took a pragmatic approach, and the primary researcher (BN) was experienced in qualitative research and aimed to build a rapport with participants before interviewing; the estimated total required sample size was around 20.

Additionally, as part of a survey collected pre‐ and post‐attendance to MECC TtT training, responses to the seven free text questions (see Data S2) included in the post‐survey evaluation (*n* = 373), which achieved a 72% response rate of all attendees across the NENC between May 2022 and 2 November 2023 (point of retrieval), were included in the qualitative analysis to increase generalizability of the findings (more details of the post‐training survey are provided in the mixed methods paper: (Nichol et al., 2026)).

### Materials and data collection

Two topic guides were developed, tailored to individuals who had or had not attended MECC TtT training. Topic guides were informed using the TDF (Cane et al., 2012; Michie et al., 2005) and pilot tested and further refined after discussion with the professional contributor panel. For principal trainers (one delivered MECC TtT not as part of the regional offer), the topic guide was applied more flexibly to reflect their different role and insight in MECC training cascade. Topic guides (see Data S2) explored the barriers and facilitators to training cascade, any existing strategies or suggestions to improve training cascade, experience of and reflections on the TtT programme and associated resources (e.g. bi‐monthly trainer forum) and thoughts on the TtT model of training delivery. For those who had not attended MECC TtT training, the topic guide focused more on their motivations to attend and barriers or facilitators to accessing MECC TtT training. Additionally, free text post‐survey questions (Data S3) centred around the training experience, whilst the interviews also asked about accessing the training and experiences in attempting to cascade MECC training after the TtT training.

After potential participants expressed interest to take part, a time and location to conduct an interview was arranged. Participants who were outside of health care were provided with the option of an in‐person or online interview via Teams, and interviews with participants within health care were conducted online. Thus, three interviews were conducted in‐person and the remaining 18 online. The primary researcher (BN) that conducted all semi‐structured interviews is female and experienced in both conducting interviews and with MECC, including previous attendance to the MECC TtT programme. Thus, the interviewer had previously met and built rapport with some participants, and built rapport with any participants where a relationship had not previously been established. The interviewer introduced themselves to all participants as a research assistant on the project. One‐to‐one interviews flexibly applied one of the two topic guides (described above). Interviews lasted between 18 and 88 min and were digitally recorded and transcribed verbatim via a transcription service and anonymized.

### Data analysis

Qualitative data (interviews and free text comments) were analysed using the Framework Method (Gale et al., 2013). To facilitate familiarization with the data, all interviews were conducted and analysed by the primary researcher (BN) through line‐by‐line coding. Namely, themes were formed deductively using the TDF domains (Cane et al., 2012); however, within each domain, inductive analysis was applied whereby codes were built into sub‐themes. Interview data and post‐survey comments were analysed separately, although analysis of the free text comments was used to inform sub‐themes identified using the interview data. Given the lack of context provided within the free text comments, their analysis was supplementary to the interview data and added to existing themes formed from the interview data. A 10% sample of survey and interview data was independently coded to ensure inter‐rater reliability of TDF coding (by SA and MYT, respectively), with disagreements resolved through discussion between all three independent raters (BN, MYT and SA). Additionally, as conducted within previous research (Lawton et al., 2015), meta‐themes were developed by the primary researcher (BN) accompanied by discussion with the core research team (CH and AMR). Meta‐themes transcended single TDF domains and were inductively created, although informed by sub‐themes and codes within each domain.

A coding system was used to label transcripts of principal trainers (PT), those who had attended the MECC TtT training (AT) and those who had not attended the MECC TtT training (NA), respectively. In accordance with open data procedures, fully anonymized transcripts were uploaded onto the UK data service (Nichol et al., n.d.‐b), a public repository, with the consent of participants using an ‘as open as possible, as closed as necessary’ principle (Society BP, 2020). Namely, given that the nature of the topic was not overly sensitive or personal for participants, it was judged appropriate to upload transcripts onto a public repository provided informed consent was attained. All data analysis was conducted via Nvivo 12 Pro (Ltd QIP, n.d.).

## RESULTS

### Themes and sub‐themes guided by the TDF

Specific barriers and facilitators according to TDF domains are described in the mixed methods paper (Nichol et al., 2026). The main sub‐themes within each TDF domain and corresponding example codes are provided in Table 1, and the full coding framework (including example quotes) is provided in Data S3. Participants placed value on individuals who were interested in MECC self‐selecting themselves to become MECC trainers as opposed to mandatory training with disinterested attendees (Intentions). However, top‐down drivers were perceived to be much more valuable. For example, a reported difficulty in identifying an audience was most often attributable to a limited staff capacity to attend MECC training (a top‐down influence) rather than a lack of interest in MECC (a bottom‐up influence). Thus, the paucity of incentives to deliver MECC training meant that benefits for MECC trainers to delivering MECC training without top‐down support was low (Reinforcement). Also, some participants suggested that ‘TtT’ is not clear on what it requires from attendees (Environmental Context and Resources), demonstrable by the finding that many attendees were unaware that they were attending a TtT programme (Knowledge) and instead wanted to learn more about MECC rather than deliver MECC training (Goals).

**TABLE 1:** Main sub‐themes within each domain and example corresponding codes from the overall coding framework Data S1. The TDF domain optimism is not included as its relevance to the data was minimal.

TDF domain	Sub‐theme	Example codes
Behavioural regulation	Monitoring and improving	Measuring cascade
Beliefs about capabilities	Confidence to deliver MECC training	Low confidence to deliver certain elements (e.g., theory content) Existing trainers are more confident in training delivery
	Perceived need and ability	Belief that staff already deliver MECC (so don't need training)
Beliefs about consequences	Beliefs around the impact of MECC	Belief in the effectiveness of MECC Believes in the value (and utility) of staff receiving MECC training
Emotion	Passion towards MECC	Passionate about and sees the value of MECC
	Emotions around training delivery	Fear of training delivery (if hadn't delivered training before)
Environmental context and resources	Top‐down drivers to support cascade	No top‐down support Top‐down support for MECC model
	Bottom‐up drivers to support cascade	Bottom‐up drivers (e.g., personal passion for MECC drives culture change)
	Engaging and mobilizing the right individuals in the right way	Expectations for cascade were not clear at sign up stage Reframing of MECC training to acknowledge staff may already be conducting MECC
	Infrastructure to support cascade at organization	Designated time to deliver training (e.g., during designated Continued Professional Development time)
	Limited time	No time to deliver or attend MECC training
	Balancing utility with feasibility	Face‐to‐face training as preferred (balance opportunities for interaction and shared learning with logistics and reach of training)
	Accessibility considerations	Difficulty in accessing (training) resources
Goals	Goals that facilitate cascade of MECC training	MECC aligns with other goals (e.g., to encourage person‐centred care)
	Goals (or lack of) to attend TtT and deliver MECC training	Lack of communication at sign up stage means that some attendees don't want to cascade MECC (only wanted to receive MECC training)
Intentions	Motivation and interest in cascading MECC training	Motivated to deliver MECC training Self‐selected to attend TtT shows intrinsic motivation, mandatory training means disengagement
Knowledge	Knowledge of the training content needed	Statistics and theory too high level (for what is needed for MECC)
Memory, attention and decision processes	Learning process during TtT training	No framing of MECC before policy and theory (leading to uncertainly of its relevance) TtT training catered to a range of learning styles
	Retaining memory after TtT training	Time delay after receipt of TtT training means content is forgotten
Reinforcement	Intrinsic or extrinsic consequents for the individual	No reward for delivery of MECC training
	Extrinsic consequents for the organization	Reward for organization (if recorded)
Skills	How to be a MECC trainer	MECC TtT insufficient for non‐trainers
	Interactive TtT training needed	No opportunity to practice training delivery
Social influences	Peer support as essential to facilitating cascade	Support from other staff at organization (e.g., discussion of tips and advice for training delivery) Peer learning (which would be helpful to continue after the TtT training)
Social/professional role and identity	Ownership of MECC training delivery as part of role	MECC trainer as part of role

On attending the MECC TtT programme, a high level of knowledge was required to deliver the MECC training content around public health statistics, health inequalities and theory (Knowledge), thus participants commonly felt a lack of confidence to deliver the content (Beliefs about Capabilities). Participants were unclear on how much of the complex information related to MECC delivery, so found it difficult to consolidate it (Memory, Attention and Decision‐Making Processes), especially if they had no experience in training delivery, which created stress or discomfort around delivering MECC training (Emotions). Face‐to‐face training encouraged facilitators, such as shared learning and interaction, but the logistical challenges of face‐to‐face training were acknowledged by participants (Environmental Context and Resources). Refresher training was thought to update knowledge, aid memory of content, revive motivation to deliver training and encourage social support (Environmental Context and Resources).

After receipt of MECC TtT training, some participants discussed their concern that staff in their organization were already competent in the skills required to deliver MECC and engaged in MECC conversations without the need for training (Beliefs about Capabilities). Subsequently, these participants expressed uncertainty of how to approach MECC training in order to acknowledge the existing competency of the audience. Also, a time delay between attending the MECC TtT programme and cascading MECC training made cascading less likely as momentum was lost (Environmental Context and Resources) and content was forgotten (Memory, Attention and Decision‐Making Processes).

### Meta‐themes that transcend TDF domains

Four meta‐themes were identified (see Table 2 for example quotes). Each meta‐theme is discussed in turn.

**TABLE 2:** Corresponding quotes for each of the four meta‐themes.

Meta‐theme	Quotes
The need for practical and psychological preparedness to deliver MECC training	AT7: ‘I think there's definitely something that's needed that then makes it actionable and I think maybes it is that kind of practical right so I've done that session on MECC what it could be within the same core session, but actually this is my little action plan that I'm going to kind of start with and then support after that’ AT6: ‘if you have somebody with a plan coming out and saying it's already been discussed, this is where we're going to go with it, and I want to deliver this, this and this and I've actually already emailed people. That's what I would have said is the best way. So, the strategy afterward, they have to be prepared to be ready to go’
Successful cascade is influenced by the MECC training content	AT11: ‘I think if you're going to ask someone to then train that and they don't have that background, if you make it too overly complicated, they're going to go all right, yeah I'll do the train the trainer and then they go nah, I don't feel confident because I don't actually understand that part myself.’ AT6: ‘I froze because I looked at the slides and went, I don't understand them. I don't get them. And when I looked and I had that conversation with my boss and I said look, I don't get it and I'm struggling with it and it's causing me stress. And she, because she's from a higher level, she was like I don't understand why, this is what you work with all the time. I says yeah, but these are heavy stats. These are Band 6. There's this more experience talking about the stats, so if there's any questions you can answer them comfortably. So, I avoided doing that. I don't think I was very popular. But it was causing me stress. It was causing me a bit of wellbeing, mental wellbeing issues’ PT1: ‘And they'd just made it their own and it was just like well that's my job done. All it took for me was you're the experts, I'm not. I'll give you these slides, but it doesn't mean anything unless you've got ownership’
Limited accessibility of the pedagogical approach to both MECC TtT and MECC training	AT11: ‘the trainers are NHS background, so by default I'm an NHS learner, sort of kind of we've all been, so regardless of your learning style, we're probably conditioned to learn in a certain way. Well, it is because NHS background's usually we'll train you, see one, do one, off you go kind of background. And I think that's what the training is like, whereas my colleague who did the training with me is then coming with me to do further, you know, to observe the training that I do, and I wonder if that's because she's not that kind of see one, do on, off you go kind of learner’ AT7: ‘Maybes it's kind of having that conversation with the team to bring them on board, help them to understand where MECC fits, but then right, well how do we do this together as a team. So, maybes it's more around rather than cascading training, maybes it's around equipping people to having facilitated conversations’ AT13: ‘some of it might have to happen in trust induction or on a pre‐existing training programme, like we've got preceptorship and care certificate, health and wellbeing champions is another one that we've got and we weave MECC into those. So they're quite good because we've got a bit of a captive audience and quite high numbers’ AT1: ‘I think what didn't work on that day for some of the team was the Train the Trainer directly following, like immediately following the core training. I don't think that really worked because as I say there were a number of people in that core MECC training who had no experience with MECC, never heard of MECC, didn't know what it was, who went through the core training and came out the other side a little bit sort of confused and bamboozled and then within 30 min later they're then going into Train the Trainer. And I just think that was too much for them on that day’
A need to shift the focus from quantity to quality of MECC training cascade	AT11: ‘I think that the blanket approach is quite often a ticky box approach of well all of our staff are trained in MECC. Lovely but have you moved that forward? Have you embedded that into policy, into working practices, into everyday activities. And I think train the trainer should be that person who's going well hang on, you know, can we do things differently… and then that can disseminate and be the voice for actually we need to do this, or we need to change this. You do need that because if you're trained in it and then there's no champion there going well can we do this, can we do that, I sometimes think it just gets well right we've done that and it's in a folder with everyone's name on and nothing happens’ PT1: ‘And they gave a total different spin on it to what I would do from a public health and they were very human and funny and you could just see how they made it their own’

#### The need for practical and psychological preparedness to deliver MECC training

From the post‐survey comments, it was apparent that many participants showed no intention of utilizing the MECC TtT programme to deliver training, with some noting their surprise at the TtT training. Many TtT attendees were not prepared to become a MECC trainer. Yet as the MECC TtT training was readily available, individuals were nonetheless encouraged to complete it. Individuals felt more ready to deliver MECC training if it helped to fulfil their role and achieve strategic goals that were set by leadership and management. Active involvement in the planning and goal setting for MECC training delivery created more motivation and preparedness to deliver MECC training than if goals were imposed onto them.

#### Successful cascade is influenced by the MECC training content

Relatedly, active involvement in tailoring the MECC training content encouraged personal ownership over the content, which facilitated personal responsibility to cascade, which was particularly helpful for trainers whose delivery of MECC training was not part of their role. Furthermore, ownership over the course content encouraged training cascade.

MECC training content could also in itself be a barrier to cascade. The high informational load of the course content meant that participants reported feelings of overwhelm and required a lot of time to familiarize themselves with it after the MECC TtT programme. Specifically, those who were newly exposed to the statistics and theory content proposed that it was overcomplicating what they were being asked to do. Also, some participants felt limited by their abilities to amend the content accordingly for worries of compromising on training quality and consistency.

#### Limited accessibility of the pedagogical approach to both MECC TtT and MECC training

Despite participants reporting that a range of learning styles were catered to within the training, a didactic training approach meant that some participants reported feelings of overwhelm at immediately learning how to deliver MECC training after receiving it themselves. Whilst participants from health care settings struggled acutely with time and capacity issues, participants within these settings tended to be familiar with the didactic training style and TtT model of training delivery, particularly those with nursing backgrounds. Thus, combined with possessing higher relevant knowledge relating to MECC and feeling MECC to be part of their role, participants within health care settings were more confident with the pedagogical approach of the MECC TtT programme, whilst participants from other settings required more support.

Considering the learning approach of the MECC training, a minority of participants discussed alternative approaches to dissemination of MECC principles. For example, informal training delivery through conversations with other staff during existing meetings was considered to be more feasible and to encourage a culture change. Others shortened MECC training to make it more accessible or delivered it as part of other training programmes in order to frame MECC. Such seemingly beneficial adaptations suggested a need to consider the definition of ‘successful’ cascade of MECC training.

#### A need to shift the focus from quantity to quality of MECC training cascade

Monitoring was mainly discussed in relation to achieving organizational level targets concerning MECC delivery, through numbers of individuals who had received TtT training, number of MECC training sessions delivered and number of individuals delivered to. Whilst easily estimating the success of the TtT model through the number of individuals trained could be motivating, it could also encourage undue focus on numbers rather than the quality of TtT training and the value of the subsequent cascaded MECC training. Combined with a wide availability of the MECC TtT training, top‐down support often encouraged attendance at the MECC TtT training but did not extend further than attendance, including the preparation, planning, and time and audience needed to cascade MECC training. Thus, there was an overall focus on individuals completing the MECC TtT training, although this was insufficient to encourage cascade.

Despite this, participants recognized that there was a unique value to the TtT model in that it encouraged trainers with knowledge of the organizations and individuals they were delivering training to, thus increasing the quality of MECC training that was delivered.

## DISCUSSION

In summary, a lack of communication about the programme, a TtT training programme that is more readily available than MECC training itself and top‐down support to attend the TtT training but not cascade MECC training means that most individuals who attend the TtT training across the NENC are not ready or willing to deliver MECC training. Furthermore, the MECC TtT programme across the NENC is relatively inflexible and inaccessible for individuals outside of health care settings and those with limited existing public health knowledge and training experience. Also, a didactic TtT training style does little to encourage ownership of MECC training for MECC trainers, which is essential in maintaining motivation to deliver training particularly when there are no incentives or expectations to cascade training. Whilst ownership of the MECC training content could be facilitated through opportunities for MECC trainers to tailor the MECC training, this flexibility was hampered by concerns of compromising the quality of MECC training and there was a lack of guidance in navigating such worries. Ironically, a focus on delivering the TtT programme as intended by monitoring ‘success’ through attendees of the TtT training and cascade rates overlooked the added value of the TtT model in encouraging MECC training that was relevant to its audience. Finally, for bottom‐up processes to become more impactful in encouraging MECC training delivery, MECC TtT training must focus on the ‘why’ and ‘how’ rather than provision of information, or the ‘what’ alone.

Many of the challenges surrounded a balance between the flexibility of MECC training (which was a strength) and the extent to which MECC training can be amended to retain its quality and consistency. However, personal ownership and buy‐in of the training content is a key factor for successful cascade (Lefkowich et al., 2018; Martinez Leal et al., 2022; Mormina & Pinder, 2018), which can be achieved through modifying the content and the teaching style and trainers adding their own experiences (Martinez Leal et al., 2022; Mormina & Pinder, 2018; Ngeze et al., 2018). The ability to amend training content in turn also increases confidence to deliver training (Martinez Leal et al., 2022). Furthermore, ownership and buy‐in to the training has been found to be accelerated through tacit knowledge (Martinez Leal et al., 2022) or experiential learning (Lefkowich et al., 2018). In contrast to the mostly didactic approach to the MECC TtT programme reported by participants, experiential learning encourages trainees to actively engage in their own learning, including through practical experience and reflection (Baker et al., 2002). For example, the healthy conversation skills (HCS) approach to MECC training focuses less on imparting public health knowledge and more on teaching trainees the skills to conduct motivational conversations (Barker et al., 2011) and has its own approach to the TtT model that mirrors the reflective HCS principles (Hollis et al., 2022). Furthermore, HCS training has been repeatedly demonstrated to encourage service providers to deliver MECC (Hollis et al., 2021; Jarman et al., 2019; Lawrence et al., 2016; Lawrence, Vogel, et al., 2020; Lawrence, Watson, et al., 2020). Consequently, the HCS TtT approach disseminates effective training content which could facilitate successful cascade itself, encouraged through an experiential learning TtT approach. Thus, the themes identified within this study appear to be tightly interconnected and support a HCS approach to delivering MECC training through the TtT model. Through facilitating buy‐in and ownership over MECC training, individuals come to see themselves as ‘MECC trainers’ (Martinez Leal et al., 2022). Findings from the current study indicate that the content of training delivered is inherently predictive of the success of the TtT model that aims to disseminate it. However, it is important to note that beyond the characteristics of the TtT training itself, organizational level support is key to determine successful cascade (Hollis et al., 2022).

Beyond its potential for greater efficiency, the TtT programme does appear to facilitate the delivery of MECC training that is relevant to the individuals it is delivered to and this asset should be fostered and encouraged. Namely, trainers with knowledge of the local area and organization could provide context to MECC that may help to increase the quality of MECC training, thus its outcomes including satisfaction of trainees and behaviour change. Indeed, in addition to cost effectiveness (Yarber et al., 2015), specific benefits of the TtT model include local knowledge (Harper et al., 2024; Yarber et al., 2015) and encouragement of collaboration across sectors (Orfaly et al., 2005), with many participants within the current study citing face‐to‐face training as an opportunity to network. Thus, there remains rationale to optimize the TtT model rather than abandon it. Resulting recommendations for optimizing the TtT programme for MECC training delivery (Nichol et al., 2026) largely reflect those in the wider education literature, including requiring that prospective MECC trainers have experience of teaching (El‐Hamamsy et al., 2024; Pearce, Jones, et al., 2012), are passionate about the topic (El‐Hamamsy et al., 2024; Hahn et al., 2002) and possess managerial support before attending the TtT programme (Pearce, Jones, et al., 2012). Following attendance at the MECC TtT programme, additional support is needed, including peer support (El‐Hamamsy et al., 2024), technical support (El‐Hamamsy et al., 2024) and follow‐up (El‐Hamamsy et al., 2024; Pearce, Jones, et al., 2012).

## STRENGTHS AND LIMITATIONS

A key strength of this study was the holistic perspective of the inclusion of those who had received MECC TtT training, were eligible but not received it, and principal trainers, individuals from across health care, local authority, and voluntary and community settings, and those who had and had not gone on to cascade MECC training. Subsequently, this created a representative sample that allowed for a comprehensive exploration into the TtT model to deliver MECC training. However, a limitation of the current study is that it focused on the NENC approach to both MECC training and the TtT programme which may limit the generalisability of the findings. Indeed, regional approaches can vary vastly including HCS (Barker et al., 2011). Whilst the findings from the current study can inform on the challenges of TtT models and the factors that facilitate successful delivery more generally including to deliver MECC training, future research would benefit from directly comparing different MECC training and TtT approaches to the delivery of MECC training, as the current study found the MECC training content to be an important barrier to successful cascade as well as the didactic TtT approach.

## IMPLICATIONS FOR PRACTICE

To facilitate successful cascade of high quality MECC training, action is needed on programme, individual and organizational levels. On a programme level, it would be helpful to reconsider the pedagogical approach to make it more accessible to non‐health care settings, including exploring more informal ways of disseminating MECC principles. It would also be helpful to review the reliance on theory and statistics within the MECC training and consider a more reflective, skills‐based approach such as HCS. On an individual level, MECC trainers should be allowed flexibility to tailor and take ownership over the MECC training content and engage in tacit and experiential learning related to how to cascade the MECC training to others. On an organizational level, top‐down ownership is needed from respective organizations that extends beyond just TtT attendance to enabling actual cascade through provision of time and resources, as is a shift of focus from quantity to quality of cascade by measuring impact rather than just numbers trained. With these changes, MECC trainers' local knowledge can be utilized to make MECC training more relevant and impactful.

## CONCLUSION

In conclusion, many factors influence perceived ownership over MECC content, which in turn facilitates MECC trainers who perceive MECC training delivery as part of their identity. The content of the MECC programme is vital for determining cascade, as is the pedagogical approach to facilitate the cascade of the content. Furthermore, more value should be placed on the quality of cascade and how this can best be achieved rather than the *‘success’* of cascade through statistics alone, to achieve the primary aim of the TtT to deliver MECC at scale.

## AUTHOR CONTRIBUTIONS

**Beth Nichol:** Conceptualization; investigation; funding acquisition; writing – original draft; methodology; visualization; writing – review and editing; software; formal analysis; project administration; data curation. **Angela M. Rodrigues:** Conceptualization; investigation; funding acquisition; writing – review and editing; validation; supervision; methodology. **Sarah Audsley:** Funding acquisition; writing – review and editing; validation. **Anna Haste:** Funding acquisition; validation; writing – review and editing. **Mei Yee Tang:** Funding acquisition; validation; writing – review and editing. **Craig Robson:** Funding acquisition; conceptualization; investigation; writing – review and editing. **Jill Harland:** Conceptualization; investigation; funding acquisition; writing – review and editing. **Catherine Haighton:** Conceptualization; investigation; funding acquisition; methodology; visualization; writing – review and editing; project administration; supervision; resources.

## FUNDING INFORMATION

This project was funded by Northumbria NHS Foundation Trust.

## CONFLICT OF INTEREST STATEMENT

BN, AMR, MYT, AH, SA and CH declare no conflicts of interest. CR and JH are the Regional MECC Coordinator and MECC Strategy Group Chair, respectively, and supported this project in terms of recruitment and data collection around the TtT programme.

## Supporting information


**Data S1.** Topic guides for (a) non‐completers of MECC TtT training and (b) completers of MECC TtT training.
**Data S2**. Post‐survey free text questions.
**Data S3**. Coding framework as guided by the TDF, with sub‐themes presented within each domain. Example codes are provided, although they are not an exhaustive list. PSC = post‐survey comments.

## Data Availability

Fully anonymised transcripts were uploaded onto the UK data service, publicly accessible here: https://reshare.ukdataservice.ac.uk/857461/. The preregistered protocol is available on Open Science Framework, publicly accessible here: https://osf.io/xz8au.

## References

[bjhp70076-bib-0001] Adam, L. M. , Jarman, M. , Barker, M. , Manca, D. P. , Lawrence, W. , & Bell, R. C. (2020). Use of healthy conversation skills to promote healthy diets, physical activity and gestational weight gain: Results from a pilot randomised controlled trial. Patient Education and Counseling, 103(6), 1134–1142. 32035738 10.1016/j.pec.2020.01.001

[bjhp70076-bib-0002] Baird, J. , Jarman, M. , Lawrence, W. , Black, C. , Davies, J. , Tinati, T. , Begum, R. , Mortimore, A. , Robinson, S. , Margetts, B. , Cooper, C. , Barker, M. , & Inskip, H. (2014). The effect of a behaviour change intervention on the diets and physical activity levels of women attending sure start Children's Centres: Results from a complex public health intervention. BMJ Open, 4(7), e005290. 10.1136/bmjopen-2014-005290 PMC4120404 25031194

[bjhp70076-bib-0003] Baker, A. C. , Jensen, P. J. , & Kolb, D. A. (2002). Conversational Learning: An Experiential Approach to Knowledge Creation. Quorum Books.

[bjhp70076-bib-0004] Barker, M. , Baird, J. , Lawrence, W. , Jarman, M. , Black, C. , Barnard, K. , Cradock, S. , Davies, J. , Margetts, B. , Inskip, H. , & Cooper, C. (2011). The Southampton initiative for health: A complex intervention to improve the diets and increase the physical activity levels of women from disadvantaged communities. Journal of Health Psychology, 16(1), 178–191. 20709878 10.1177/1359105310371397 PMC3685267

[bjhp70076-bib-0005] Bhaskar, R. (2010). Reclaiming REALITY: A Critical Introduction to Contemporary Philosophy. Routledge.

[bjhp70076-bib-0006] Cane, J. , O'Connor, D. , & Michie, S. (2012). Validation of the theoretical domains framework for use in behaviour change and implementation research. Implementation Science, 7(1), 1–17. 10.1186/1748-5908-7-37 PMC3483008 22530986

[bjhp70076-bib-0007] Chisholm, A. , Byrne‐Davis, L. , Peters, S. , Beenstock, J. , Gilman, S. , & Hart, J. (2020). Online behaviour change technique training to support healthcare staff ‘make every contact count’. BMC Health Services Research, 20(1), 1–11. 10.1186/s12913-020-05264-9 PMC7206818 32380982

[bjhp70076-bib-0008] Dahlgren, G. , & Whitehead, M. (1991). Policies and strategies to promote social equity in health.

[bjhp70076-bib-0009] Department of Health SS, Safety P . (2011). Transforming Your Care: A Review of Health and Social Care in Northern Ireland. Department of Health, Social Services and Public Safety Belfast.

[bjhp70076-bib-0010] El‐Hamamsy, L. , Monnier, E.‐C. , Avry, S. , Chessel‐Lazzarotto, F. , Liégeois, G. , Bruno, B. , Zufferey, J. D. , & Mondada, F. (2024). An adapted cascade model to scale primary school digital education curricular reforms and teacher professional development programs. Education and Information Technologies, 29(9), 10391–11436.

[bjhp70076-bib-0011] Fitzsimmons‐Craft, E. E. , Bohon, C. , Wilson, G. T. , Jo, B. , Mondal, S. , Laing, O. , Welch, R. R. , Raghavan, R. , Proctor, E. K. , Agras, W. S. , & Wilfley, D. E. (2021). Maintenance of training effects of two models for implementing evidence‐based psychological treatment. Psychiatric Services, 72(12), 1451–1454. 34189934 10.1176/appi.ps.202000702 PMC8941627

[bjhp70076-bib-0012] Gale, N. K. , Heath, G. , Cameron, E. , Rashid, S. , & Redwood, S. (2013). Using the framework method for the analysis of qualitative data in multi‐disciplinary health research. BMC Medical Research Methodology, 13(1), 1–8. 24047204 10.1186/1471-2288-13-117 PMC3848812

[bjhp70076-bib-0013] Hahn, E. J. , Noland, M. P. , Rayens, M. K. , & Christie, D. M. (2002). Efficacy of training and fidelity of implementation of the life skills training program. Journal of School Health, 72(7), 282–287. 12357909 10.1111/j.1746-1561.2002.tb01333.x

[bjhp70076-bib-0014] Harper, M. , Gore, J. , & Harris, J. (2024). A new conceptual framework for understanding and implementing train‐the‐trainer professional development on a large scale. Professional Development in Education, 51, 1–12.

[bjhp70076-bib-0015] Harrison, D. , Wilson, R. , Graham, A. , Brown, K. , Hesselgreaves, H. , & Ciesielska, M. (2022). Making every contact count with seldom‐heard groups? A qualitative evaluation of voluntary and community sector (VCS) implementation of a public health behaviour change programme in England. Health & Social Care in the Community, 30(5), e3193–e3206. 35218264 10.1111/hsc.13764 PMC9544506

[bjhp70076-bib-0016] Hayes, D. (2000). Cascade training and teachers' professional development. ELT Journal, 54(2), 135–145.

[bjhp70076-bib-0017] Hollis, J. L. , Kocanda, L. , Seward, K. , Collins, C. , Tully, B. , Hunter, M. , Foureur, M. , Lawrence, W. , MacDonald‐Wicks, L. , & Schumacher, T. (2021). The impact of healthy conversation skills training on health professionals' barriers to having behaviour change conversations: A pre‐post survey using the theoretical domains framework. BMC Health Services Research, 21(1), 1–13. 34452634 10.1186/s12913-021-06893-4 PMC8394191

[bjhp70076-bib-0018] Hollis, J. L. , Seward, K. , Kocanda, L. , Collins, C. E. , Tully, B. , Brett, K. , Hunter, M. , Foureur, M. , Schumacher, T. , Lawrence, W. , & MacDonald‐Wicks, L. (2022). Evaluating a train‐the‐trainer model for scaling‐up healthy conversation skills training: A pre‐post survey using the theoretical domains framework. Patient Education and Counseling, 105(10), 3078–3085. 35779983 10.1016/j.pec.2022.06.011

[bjhp70076-bib-0019] Jarman, M. , Adam, L. , Lawrence, W. , Barker, M. , & Bell, R. C. (2019). Healthy conversation skills as an intervention to support healthy gestational weight gain: Experience and perceptions from intervention deliverers and participants. Patient Education and Counseling, 102(5), 924–931. 30598358 10.1016/j.pec.2018.12.024 PMC7617156

[bjhp70076-bib-0020] Lang, J. , Cluff, L. , Rineer, J. , Brown, D. , & Jones‐Jack, N. (2017). Building capacity for workplace health promotion: Findings from the work@ health® train‐the‐trainer program. Health Promotion Practice, 18(6), 902–911. 28829622 10.1177/1524839917715053 PMC5735412

[bjhp70076-bib-0021] Lawrence, W. , Black, C. , Tinati, T. , Cradock, S. , Begum, R. , Jarman, M. , Pease, A. , Margetts, B. , Davies, J. , Inskip, H. , Cooper, C. , Baird, J. , & Barker, M. (2016). ‘Making every contact count’: Evaluation of the impact of an intervention to train health and social care practitioners in skills to support health behaviour change. Journal of Health Psychology, 21(2), 138–151. 24713156 10.1177/1359105314523304 PMC4678584

[bjhp70076-bib-0022] Lawrence, W. , Vogel, C. , Strömmer, S. , Morris, T. , Treadgold, B. , Watson, D. , Hart, K. , McGill, K. , Hammond, J. , Harvey, N. C. , Cooper, C. , Inskip, H. , Baird, J. , & Barker, M. (2020). How can we best use opportunities provided by routine maternity care to engage women in improving their diets and health? Maternal & Child Nutrition, 16(1), e12900. 31736283 10.1111/mcn.12900 PMC7038869

[bjhp70076-bib-0023] Lawrence, W. , Watson, D. , Barker, H. , Vogel, C. , Rahman, E. , & Barker, M. (2020). Meeting the UK Government's prevention agenda: Primary care practitioners can be trained in skills to prevent disease and support self‐management. Perspectives in Public Health, 142, 158–166. 10.1177/1757913920977030 PMC9047100 33588652

[bjhp70076-bib-0024] Lawton, R. , Heyhoe, J. , Louch, G. , Ingleson, E. , Glidewell, L. , Willis, T. A. , McEachan, R. R. , & Foy, R. (2015). Using the theoretical domains framework (TDF) to understand adherence to multiple evidence‐based indicators in primary care: A qualitative study. Implementation Science, 11, 1–16. 10.1186/s13012-016-0479-2 PMC4977705 27502590

[bjhp70076-bib-0025] Lefkowich, M. , Richardson, N. , Brennan, L. , Lambe, B. , & Carroll, P. (2018). A process evaluation of a training of trainers (TOT) model of men's health training. Health Promotion International, 33(1), 60–70. 27476866 10.1093/heapro/daw056

[bjhp70076-bib-0026] Lloyd, B. , Rychetnik, L. , Maxwell, M. , & Nove, T. (2009). Building capacity for evidence‐based practice in the health promotion workforce: Evaluation of a train‐the‐trainer initiative in NSW. Health Promotion Journal of Australia, 20(2), 151–154. 19642965 10.1071/he09151

[bjhp70076-bib-0027] Ltd QIP Nvivo. 12 Pro ed2018.

[bjhp70076-bib-0028] Malterud, K. , Siersma, V. D. , & Guassora, A. D. (2016). Sample size in qualitative interview studies: Guided by information power. Qualitative Health Research, 26(13), 1753–1760. 26613970 10.1177/1049732315617444

[bjhp70076-bib-0029] Martinez Leal, I. , Martinez, J. , Britton, M. , Chen, T. A. , Correa‐Fernández, V. , Kyburz, B. , Nitturi, V. , Obasi, E. M. , Drenner, K. , Williams, T. , Casey, K. , Carter, B. J. , & Reitzel, L. R. (2022). Collaborative learning: A qualitative study exploring factors contributing to a successful tobacco cessation train‐the‐trainer program as a community of practice. International Journal of Environmental Research and Public Health, 19(13), 7664. 35805323 10.3390/ijerph19137664 PMC9266255

[bjhp70076-bib-0030] Martino, S. , Ball, S. A. , Nich, C. , Canning‐Ball, M. , Rounsaville, B. J. , & Carroll, K. M. (2011). Teaching community program clinicians motivational interviewing using expert and train‐the‐trainer strategies. Addiction, 106(2), 428–441. 20925684 10.1111/j.1360-0443.2010.03135.x PMC3017235

[bjhp70076-bib-0031] Michie, S. , Johnston, M. , Abraham, C. , Lawton, R. , Parker, D. , & Walker, A. (2005). Making psychological theory useful for implementing evidence based practice: A consensus approach. BMJ Quality and Safety, 14(1), 26–33. 10.1136/qshc.2004.011155 PMC1743963 15692000

[bjhp70076-bib-0032] Mills, A. (2019). Helping students to self‐care and enhance their health‐promotion skills. British Journal of Nursing, 28(13), 864–867. 31303044 10.12968/bjon.2019.28.13.864

[bjhp70076-bib-0033] Mormina, M. , & Pinder, S. (2018). A conceptual framework for training of trainers (ToT) interventions in global health. Globalization and Health, 14, 1–11. 30348183 10.1186/s12992-018-0420-3 PMC6198384

[bjhp70076-bib-0034] Mounce, H. (2002). The Two Pragmatisms: From Peirce to Rorty. Routledge.

[bjhp70076-bib-0035] National Health Service N . (2014). Five Year Forward View. NHS England.

[bjhp70076-bib-0036] Nexø, M. A. , Kingod, N. R. , Eshøj, S. H. , Kjærulff, E. M. , Nørgaard, O. , & Andersen, T. H. (2024). The impact of train‐the‐trainer programs on the continued professional development of nurses: A systematic review. BMC Medical Education, 24(1), 30. 38178050 10.1186/s12909-023-04998-4 PMC10768131

[bjhp70076-bib-0037] Ngeze, L. V. , Khwaja, U. , & Iyer, S. (2018). Cascade Model of Teacher Professional Development: Qualitative Study of the Desirable Characteristics of Secondary Trainers and Role of Primary Trainers. Proceeding at the 26th International Conference on Computers In Education.

[bjhp70076-bib-0038] Nichol, B. , Kemp, E. , Wilson, R. , Rodrigues, A. M. , Hesselgreaves, H. , Robson, C. , & Haighton, C. (2024). Establishing an updated consensus on the conceptual and operational definitions of making every contact count (MECC) across experts within research and practice internationally: A Delphi study. Public Health, 230, 29–37. 38484623 10.1016/j.puhe.2024.01.023

[bjhp70076-bib-0039] Nichol, B. , Rodrigues, A. M. , & Haighton, C. Transcripts of those who attended and had not attended the making every contact count (MECC) train the trainer programme in the north east and North Cumbria. In: Service UD, editor. Colchester, Essex2024.

[bjhp70076-bib-0040] Nichol, B. , Rodrigues, A. M. , Tang, M. Y. , Haste, A. , Audsley, S. , Robson, C. , Harland, J. , & Haighton, C. (2026). Mixed methods evaluation of the “train the trainer” model for delivering core “making every contact count” (MECC) training. International Journal of Behavioral Medicine, 1–25. 41922657 10.1007/s12529-026-10444-8

[bjhp70076-bib-0042] Orfaly, R. A. , Frances, J. C. , Campbell, P. , Whittemore, B. , Joly, B. , & Koh, H. (2005). Train‐the‐trainer as an educational model in public health preparedness. Journal of Public Health Management and Practice, 11(6), S123–S127. 10.1097/00124784-200511001-00021 16205531

[bjhp70076-bib-0043] Organization WH . (2021). Saving lives, spending less: the case for investing in noncommunicable diseases.

[bjhp70076-bib-0044] Parchment, A. , Lawrence, W. , Perry, R. , Rahman, E. , Townsend, N. , Wainwright, E. , & Wainwright, D. (2021). Making every contact count and healthy conversation skills as very brief or brief behaviour change interventions: A scoping review. Journal of Public Health, 31, 1017–1034.

[bjhp70076-bib-0045] Parchment, A. , Lawrence, W. , Rahman, E. , Townsend, N. , Wainwright, E. , & Wainwright, D. (2022). How useful is the making every contact count healthy conversation skills approach for supporting people with musculoskeletal conditions? Journal of Public Health, 30, 1–17. 35530417 10.1007/s10389-022-01718-y PMC9067897

[bjhp70076-bib-0046] Parchment, A. , Lawrence, W. , Rahman, E. , Townsend, N. , Wainwright, E. , & Wainwright, D. (2023). ‘I can feel myself coming out of the rut’: A brief intervention for supporting behaviour change is acceptable to patients with chronic musculoskeletal conditions. BMC Musculoskeletal Disorders, 24(1), 1–12. 36991425 10.1186/s12891-023-06336-7 PMC10050805

[bjhp70076-bib-0047] Pearce, J. , Jones, C. , Morrison, S. , Olff, M. , van Buschbach, S. , Witteveen, A. B. , Williams, R. , Orengo‐García, F. , Ajdukovic, D. , Aker, A. T. , Nordanger, D. , Lueger‐Schuster, B. , & Bisson, J. I. (2012). Using a delphi process to develop an effective train‐the‐trainers program to train health and social care professionals throughout Europe. Journal of Traumatic Stress, 25(3), 337–343. 22648660 10.1002/jts.21705

[bjhp70076-bib-0048] Pearce, J. , Mann, M. K. , Jones, C. , Van Buschbach, S. , Olff, M. , & Bisson, J. I. (2012). The most effective way of delivering a train‐the‐trainers program: A systematic review. Journal of Continuing Education in the Health Professions, 32(3), 215–226. 23173243 10.1002/chp.21148

[bjhp70076-bib-0049] Poitras, M.‐E. , Belanger, E. , Vaillancourt, V. T. , Kienlin, S. , Körner, M. , Godbout, I. , Bernard‐Hamel, J. , O'Connor, S. , Blanchette, P. , Khadhraoui, L. , Sawadogo, J. , Massougbodji, J. , Zomahoun, H. T. V. , Gallani, M. C. , Stacey, D. , & Légaré, F. (2021). Interventions to improve trainers' learning and behaviors for educating health care professionals using train‐the‐trainer method: A systematic review and meta‐analysis. Journal of Continuing Education in the Health Professions, 41(3), 202–209. 34292260 10.1097/CEH.0000000000000375

[bjhp70076-bib-0050] Public Health England P . (2016). Making Every Contact Count (MECC): Consensus Statement.

[bjhp70076-bib-0051] Qualtrics . (2013). Qualtrics. Provo, Utah, USA.

[bjhp70076-bib-0052] Rodrigues, A. M. , Nichol, B. , Wilson, R. , Charlton, C. , Gibson, B. , Finch, T. , Haighton, C. , Maniatopoulos, G. , Giles, E. , Harrison, D. , Orange, D. , Robson, C. , & Harland, J. (2024). Mapping regional implementation of ‘making every contact count’: Mixed methods evaluation of implementation stage, strategies, barriers and facilitators of implementation. BMJ Open, 14(7), e084208. 10.1136/bmjopen-2024-084208 PMC11268057 39038864

[bjhp70076-bib-0053] Scotland N . (2013). A Route Map to the 2020 Vision for Health and Social Care. NHS Scotland.

[bjhp70076-bib-0054] Society BP . (2020). Position Statement: Open Data.

[bjhp70076-bib-0055] Tinati, T. , Lawrence, W. , Ntani, G. , Black, C. , Cradock, S. , Jarman, M. , Pease, A. , Begum, R. , Inskip, H. , Cooper, C. , Baird, J. , & Barker, M. (2012). Implementation of new healthy conversation skills to support lifestyle changes–what helps and what hinders? Experiences of sure start Children's Centre staff. Health & Social Care in the Community, 20(4), 430–437. 22452549 10.1111/j.1365-2524.2012.01063.x PMC3679516

[bjhp70076-bib-0056] Tobias, C. R. , Downes, A. , Eddens, S. , & Ruiz, J. (2012). Building blocks for peer success: Lessons learned from a train‐the‐trainer program. AIDS Patient Care and STDs, 26(1), 53–59. 22103430 10.1089/apc.2011.0224 PMC3242619

[bjhp70076-bib-0057] Tong, A. , Sainsbury, P. , & Craig, J. (2007). Consolidated criteria for reporting qualitative research (COREQ): A 32‐item checklist for interviews and focus groups. International Journal for Quality in Health Care, 19(6), 349–357. 17872937 10.1093/intqhc/mzm042

[bjhp70076-bib-0058] Trabeau, M. , Neitzel, R. , Meischke, H. , Daniell, W. E. , & Seixas, N. S. (2008). A comparison of “train‐the‐trainer” and expert training modalities for hearing protection use in construction. American Journal of Industrial Medicine, 51(2), 130–137. 18067179 10.1002/ajim.20499

[bjhp70076-bib-0059] Turner, R. , Byrne‐Davis, L. , Michael, P. , Coupe, N. , Holtom, C. , Smith, C. , & Hart, J. (2023). Experiences of implementing the ‘making every contact Count’ initiative into a UK integrated care system: An interview study. Journal of Public Health, 45, 894–903. 37717953 10.1093/pubmed/fdad173 PMC10689001

[bjhp70076-bib-0060] Wales N . (2011). Together for Health: A Five Year Vision for the NHS in Wales. Welsh Government.

[bjhp70076-bib-0061] Watson, D. , Godfrey, P. , Rahman, E. , Varkonyi‐Sepp, J. , & Lawrence, W. (2020). Adapting making every contact count/healthy conversation skills to pilot online supportive conversations training in response to Covid‐19. University of Southampton Institutional Repository, University of Southampton.

[bjhp70076-bib-0062] WHO . (2015). People‐centred and integrated health services: an overview of the evidence: interim report.

[bjhp70076-bib-0063] World Health Organization W Self‐care for health and well‐being 2024. https://www.who.int/news‐room/fact‐sheets/detail/self‐care‐health‐interventions

[bjhp70076-bib-0064] Yarber, L. , Brownson, C. A. , Jacob, R. R. , Baker, E. A. , Jones, E. , Baumann, C. , Deshpande, A. D. , Gillespie, K. N. , Scharff, D. P. , & Brownson, R. C. (2015). Evaluating a train‐the‐trainer approach for improving capacity for evidence‐based decision making in public health. BMC Health Services Research, 15, 1–10. 26652172 10.1186/s12913-015-1224-2 PMC4676893

